# The Evolution of Protein Structures and Structural Ensembles Under Functional Constraint

**DOI:** 10.3390/genes2040748

**Published:** 2011-10-28

**Authors:** Jessica Siltberg-Liberles, Johan A. Grahnen, David A. Liberles

**Affiliations:** Department of Molecular Biology, University of Wyoming, Laramie, WY 82071, USA; E-Mail: jgrahnen@uwyo.edu

**Keywords:** conformational ensemble, multiscale modeling, structural disorder, sequence-structure-function-evolution relationships

## Abstract

Protein sequence, structure, and function are inherently linked through evolution and population genetics. Our knowledge of protein structure comes from solved structures in the Protein Data Bank (PDB), our knowledge of sequence through sequences found in the NCBI sequence databases (http://www.ncbi.nlm.nih.gov/), and our knowledge of function through a limited set of *in-vitro* biochemical studies. How these intersect through evolution is described in the first part of the review. In the second part, our understanding of a series of questions is addressed. This includes how sequences evolve within structures, how evolutionary processes enable structural transitions, how the folding process can change through evolution and what the fitness impacts of this might be. Moving beyond static structures, the evolution of protein kinetics (including normal modes) is discussed, as is the evolution of conformational ensembles and structurally disordered proteins. This ties back to a question of the role of neostructuralization and how it relates to selection on sequences for functions. The relationship between metastability, the fitness landscape, sequence divergence, and organismal effective population size is explored. Lastly, a brief discussion of modeling the evolution of sequences of ordered and disordered proteins is entertained.

## 1. Introduction

The links between gene sequence, protein structure, and biological function are central to the development of a mechanistic understanding of molecular and cellular biological processes. Further, from an evolutionary perspective, changes in gene sequences, as filtered by protein structure and function, can drive phenotypic change through neutral and adaptive mechanisms. Selection can ultimately occur at the level of the fitness of the individual organism, filtered through the lens of cell biology down to the level of protein function, structure, and sequence. Not all proteins contribute equally to organismal fitness. The generation of high throughput genomic, proteomic, and structural datasets has enabled molecular evolutionary analysis of functional data. Ultimately, an understanding of the interplay of protein structure with both sequence evolution and functional/phenotypic evolution is necessary. This review will depict this understanding from several key perspectives.

## 2. Protein Structure Space

The nature of protein structure space is an important starting point for characterizing the link between sequence, structure, and function. Knowing how well protein structure space has been characterized (the degree to which the sampling is complete) is a necessary prerequisite for, understanding how it has evolved, the constraints on its evolution, and the constraints that it (and evolutionarily accessible alternatives) place on sequences and functions.

*That protein structure is more conserved than sequence* is a common perception among molecular life scientists. This is based upon an observation of the experimentally determined protein structures in the Protein Data Bank (PDB). However, if we remove the 100% identical proteins from PDB, we are left with about 40,000 PDB structures. If we compare that to the number of protein sequences in the RefSeq database (currently >10 million protein sequences), it is clear that our current knowledge of protein structure space is derived from a very small subset of proteins. This is especially true if it is the case that structure can vary among homologous proteins from different species with correspondingly more variation in structure than is sometimes appreciated. It is known that the protein composition of PDB is biased [1]. Membrane proteins and structurally disordered proteins are underrepresented in PDB and many proteins are modified (truncated and/or mutated) in order to facilitate crystal formation. Some proteins in fact show the hallmarks of crystal packing forces in their structures that cannot reasonably be expected to reflect that stable structure in solution [2]. There are also biases in the function, subcellular localization and protein coverage in PDB [1].

Despite these caveats, there are a lot of important data and trends to be found in the PDB. Protein structure classification, for instance CATH [3], further characterizes most multidomain structures in PDB at the domain level, as the domain is commonly regarded to be the smallest functional unit that can fold by itself. CATH currently has almost 1,300 different topologies or folds, some of which are used much more frequently than other folds. However, while this data is focused on the domain level, it misses structural organization at the multidomain level. Many multidomain proteins contain linker sequences between domains and the structural flexibility of these linkers has informational value for our understanding of the extended protein structure space. If we can estimate the extent of structural flexibility between domains, it would certainly add to the current understanding of how protein structures evolve on the tertiary and quaternary structural levels. Not only could intra-chain domain-domain packing be affected in the tertiary structure, but also inter-chain domain-domain packing can be affected in (for instance) the case of domain swaps. These studies are likely to increase our understanding of how domain-domain crosstalk and allostery evolve, which can improve current methods for homology modeling of multidomain complexes and correspondingly, our understanding of the evolution of protein function, interaction, and regulation.

**Figure 1:**
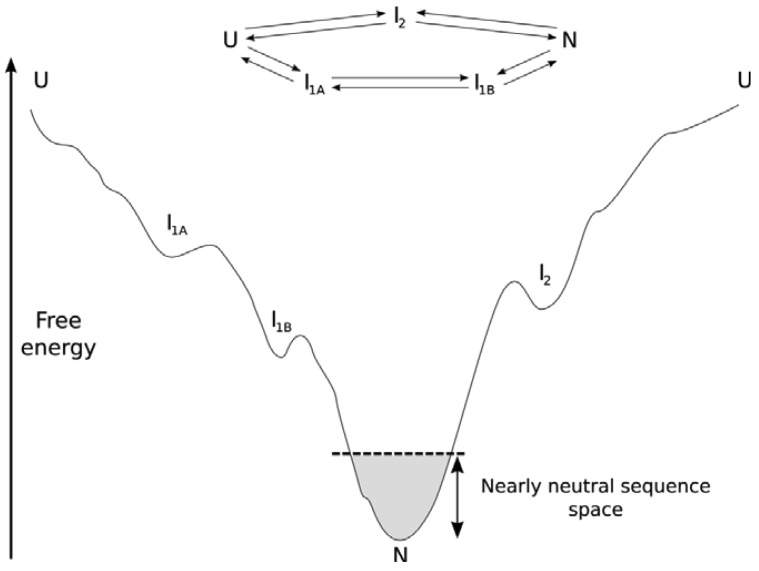
A possible conformational energy landscape for a typical structured protein. The protein has two alternative folding pathways (top), proceeding from the unfolded state (U) to the native state (N) through one (I2) or two (I1A, I1B) intermediate conformations. The funnel-shaped landscape guarantees rapid folding to the native state, passing various metastable states with different rates of interconversion on the way. The shaded area near the native state indicates the magnitude of change in folding energy that is selectively neutral (dependent upon to population size Ne and selective pressure s).

Focusing on the PDB as our source for protein structure information may lead us to a skewed view of protein structure space. From a structural rather than a functional perspective, proteins that rarely make it into the PDB simply because they are too dynamic are systematically missed. Due to the nature of the energy landscape (the relative energies of different conformations and ultimately different sequences in different conformations, see Figure 1), these are the proteins that exist in rapidly exchanging conformations and that may only progress down the folding funnel towards a stable conformation after being either post-translationally modified or when interacting with a binding partner. These proteins are commonly referred to as structurally disordered. Structurally disordered proteins can be fully or partially disordered, and what is intriguing about these proteins is their presence as a conformational ensemble that kinetically interconverts on cellular timescales. Here we cannot simply say that protein structure is more conserved than sequence because a mutation in the conformational ensemble is likely to shift the equilibrium of the conformational ensembles. Hence, the evolution of structurally disordered proteins may lead to non-conserved protein structures among homologs through this shift in the conformational ensemble. We call this phenomenon neostructuralization [4]. Starting with the structurally ordered proteins, we will attempt to systematically describe our understanding of how proteins evolve. As our understanding of protein sequence-structure links and the intertwining roles of physical chemistry and evolution improves, key aspects of our knowledge based on protein structural evolution may need revision.

## 3. Evolution of Structurally Ordered Proteins

Structured domains are characterized by a large proportion of secondary structure, as well as a single hydrophobic core and mostly hydrophilic surface. Distinct regions of non-enzymatic proteins with different evolutionary properties include the hydrophilic surface, the hydrophobic core, and more hydrophobic surface binding interfaces involved in protein-protein interaction. These regions show different rates of amino acid substitution, with the hydrophobic core evolving more slowly than the hydrophilic surface [5]. Quantitatively, core residues evolve up to 10x slower than surface residues [6], and include residues that are the most informative for determining the topology of the native fold [7]. In fact, rates of evolution correlate strongly with fractional residue burial [8]. Within protein families, backbone change in the core increases very slowly [9], mostly preserving the characteristic topology of the fold over relatively long evolutionary distances. Single substitutions are generally accommodated by side chain packing [10]. The structure dictates the inter-residue interactions that occur and the thermodynamic intramolecular coupling of substitutions is detectable from evolutionary data [11], leading to the use of contact maps and viewing proteins in a network context [12]. For proteins with a binding function, the binding interface is under functional constraint and may evolve the slowest, with differences in rate between affinity-determining and specificity-determining residues [13]. Different secondary structural elements also show different rates of evolution, with beta-sheet regions evolving more slowly than helical regions, and with random coil regions evolving fastest [5,14]. Beyond secondary structure, this may be influenced by differences in relative burial between different elements. In addition to point substitutions, insertion and deletion events (indels) also occur at varying rates [15].

While it sometimes supposed that Hidden Markov Model (HMM) emission probabilities from Pfam [16] reflect the allowed nature of sequence divergence within a structure and describe aspects of allowable sequences within structures, these have been generated without consideration of the phylogenetic scale on which sequences have been diverging. Kondrashov [17] has suggested that explored sequence space within folds of real proteins is still expanding. Consistent with this, evolutionary simulation imply that there are many sequences that have not been observed that can fold into a given known structure [18,19]. This is also consistent with observations from protein design [20–22]. These views may necessitate revision of our understanding of the uniqueness of superfolds and related concepts of designability, leading to alternative hypotheses for fold distributions rooted in evolutionary and population genetic processes [23–25].

For the subset of proteins that form a stable unique tertiary structure, the thermodynamic stability (ΔG) of the protein in the context of a folding funnel is important [26] (see Figure 1). It is therefore maintained throughout evolution despite the average destabilizing effect of non-synonymous mutations [27–29]. Proteins are only marginally stable, with a free-energy change of a few kcal/mol upon folding [30]. This has been attributed to population-level neutral processes, where there is more power to select for a larger energy gap in larger population species (organisms) or when there is a strong selective advantage to do so (as in hyperthermophiles) [31,32], or alternatively to functional requirements for protein flexibility [33]. To overcome the Levinthal Paradox, distal parts of the energy landscape must be gently sloping towards the native structure(s). However, the metastability of the folded structure relative to alternative folded structures combined with dN/dS data suggesting strong negative selection on the average protein against the average mutation [5] suggests that the local funnel near the native state is more rugged from a mutational perspective through evolution than other parts of the landscape, with allowable mutations forming a neutral network. Ultimately, structure is important as a scaffold for properly orienting functional residues (for example, a binding interface, catalytic residues, or a pore). Consequently, there is little selective pressure for particular sequences within a given structure over longer evolutuionary periods, generating a neutral network of sequences connected by those accessible through the mutational process. Folds with excess ΔG are thought to possess more potential for neofunctionalization (and gene family expansion [30,34–36]. But as expected from nearly-neutral theory [37], the majority of mutations are either deleterious or neutral rather than adaptive, both in terms of ΔG and fitness [27,29,38–40]. Compensatory mutation can play a selective role within nearly neutral sequence networks, whereby a deleterious mutation makes a subsequent otherwise neutral change selectively advantageous [9].

The processes described above can lead to structural transitions through two different processes. Within a neutral network that is functional, there may be multiple structural states that can exist. It is unclear that there is always a selective pressure for an energy gap near the native structure(s), especially in the case that closely related structures are functionally equivalent. Changes in secondary structure content after residue substitutions can occur due to varying helix/sheet propensity, with sheets being more plastic [5,14]. Some of these changes in secondary structural composition are likely to be evolutionarily neutral. A second mode of structural transition involves positive selection. In this case, a new fold that is mutationally accessible may enable the development of a new function that was not possible within the previous fold.

This raises an interesting question: is protein structure space continuous or discrete in enabling evolutionary transitions between distinct folds? A variety of measures of structural similarity have been applied to construct maps of protein structure space [41–43]. These maps consistently show highly populated regions roughly corresponding to the Class level of SCOP [43–45], and smaller clusters corresponding to the presumably homologous Superfamily level [43]. Depending on the algorithms and graph-theoretical measures employed, different groups have argued that this space is fully connected [42] or highly fragmented [43]. However, mechanistically protein evolution does not proceed via jumps in structural space/geometry as it is sometimes modeled, but via small changes in sequence space and the mapping between structural hierarchies and mutation-based hierarchies is unclear. While circular permutation and other larger scale mutational re-arrangements have been observed [46], the important consideration is that fold transitions occur through the mutational process at the sequence level rather than geometrically at the structural level as it is sometimes modeled. To rigorously evaluate the possibility of a fold transition one would have to determine the viability of a series of mutations that connect the two folds. Both thermodynamics and kinetics of folding must be taken into account, as well as fitness effects due to function, all within in a context of population genetics.

## 4. Evolution of Protein Folding Pathways

In addition to a unique and stable native state, structured proteins also have pathways through which they rapidly fold. In some cases, the folding pathway has been shown to affect the final structure that the sequence folds into, meaning that the folding pathway can be important to the ultimate fold and therefore the ultimate biological function (for example, [47]). It is only to the extent that folding pathway effects structure and ultimately function that it is evolutionarily important. Folding pathways do also have an important role in preventing aggregation, with proper folding driven at least partly by hydrophobic collapse. With these views in mind, the conservation of folding pathways is described.

The intermediates in the folding pathway are known to be conserved for some homologous proteins [48]. The correlation between native state contact order and folding kinetics [49] further suggests that the native state topology is the main evolutionary determinant of the folding pathway. A number of studies [50,51] subsequently showed that folding pathways are partially, but not fully, conserved in homologs of single-domain proteins. Folded subdomains (folding nuclei or foldons) can be strongly conserved, particularly if they define an intermediate or transition state late in the pathway [52,53]. However, even very small proteins appear to have multiple parallel pathways and intermediates [53,54], and the flux through each pathway can change appreciably after mutation [52,55,56]. Earlier stages of folding appear to be less conserved than later stages [57]. Variability in the ruggedness of the energy landscape containing a folding funnel [26] depending upon distance from the native state can explain these observations. In the early stages of folding, the funnel is very wide and multiple pathways may lead into it over a variety of transition states. As the bottom of the funnel is approached (for the classic funnel model with a single minimum), the width (*i.e.*, number of available conformations) shrinks and fewer pathway options exist for proteins with a single native state. Additionally, as the number of native contacts increases, the choice of pathways becomes increasingly dominated by the topology of the native state, including specific residue contacts [50,58]. The early and intermediate conformations are stabilized by various non-native contacts, which do not contribute to the stability of the fully folded state and are therefore under less selective pressure to be maintained within a fold if they are not necessary for proper folding. Ultimately, the shape of the folding funnel near the folded conformation and towards the edges of the native sequence landscape in the context of marginal stability is an open question, as is the existence of divergent structures dependent upon folding pathway for some protein families.

## 5. Evolution of Conformational Ensembles and of Protein Dynamics

Given the potential continuity of fold space and of the underlying sequence space, it is clear that proteins can exist in conformational ensembles, both functionally and as evolutionary transitions. Beyond thermodynamic considerations of conformational ensembles is the role of kinetics in protein structure and function. This section will focus on the motion of individual proteins.

As a neutral baseline, Illergard *et al.* [6] established an approximately linear divergence between the rate of sequence evolution and of structural divergence measured by structural root mean square deviation (RMSD) evolution for static structures. There is a relationship between the lowest energy normal modes and the paths through which protein structure diverges through mutational opportunity [59]. Further, it has been established that the lowest normal modes also evolved with approximate rate of divergence proportionality to the structural divergence hierarchy [60,61]. Deviations from this clock-like rate may be expected to show a functional signal that may evolve particularly rapidly under processes like positive selective pressure. The hypothesis, then, is that rate accelerations in normal mode divergence may be useful in predicting functional divergence.

A confounding factor is the role of post-translational modification in modifying thermodynamic and kinetic conformational ensemble stabilities, especially as patterns of post-translational modification can evolve rapidly on evolutionary timescales. As will be discussed further below, post-translational modification can alter the equilibrium in a conformational ensemble and may therefore play a more major role than is commonly attributed in protein structure determination. From an evolutionary perspective, selection on folding stability and pathway may interplay with selection on sites for post-translational modification.

Given that ensembles of structures can play functional roles and can be found as either evolutionary intermediates or as evolutionarily stable functional proteins, the question emerges, how do these proteins that are disordered or in rapidly shifting equilibria between ordered structures evolve?

## 6. Evolution of Structurally Disordered Proteins

Study of the evolution of structurally disordered proteins is in its infancy. It has been predicted that the fraction of structurally disordered protein increases with organismal complexity [62], but why is unclear. This may be linked to the increase in the frequency of multidomain proteins with organismal complexity [63,64]. An increase in multidomain proteins also means more domain spacers or linkers, which often are structurally flexible. More fundamentally, more complex organisms (as defined by the number of distinct cell types) tend to have smaller population sizes and reduced strengths of selection. A null hypothesis for the rise of disorder in these lineages might simply be a reduction in the strength of selection along these lineages, including on proper protein folding [65]. To reject this hypothesis, we would need to detect selectable functions in disordered proteins that cannot be accomplished by ordered proteins. Fundamentally, we would also need to account for the ability to select for these features in evolutionary regimes where selection has less power, such as in small population size multicellular animals.

To understand how structural disorder evolves and if it is conserved or not, one needs to study the evolutionary dynamics of disordered regions in the phylogenetic context of homologous proteins. Studies of this kind are scarce, but it appears that structural order and disorder, as well as underlying secondary structural propensities, are conserved in some homologs, but not all [4]. For example, patterns of disorder, like other evolutionary features, appear more likely to shift among paralogs than among orthologs [4]. Further studies are needed to characterize these trends in greater detail. Like structured proteins, different structurally disordered proteins evolve at different rates, although there is a tendency for structurally disordered regions to evolve at higher amino acid substitution rates than structured proteins [66–68]. A recent effort to calculate a disordered protein specific substitution matrix also shows that specific matrices for these proteins can be generated [69] but unfortunately, the generality of such matrices is dependent upon the conservation of selective pressures within disordered regions and the conservation of disorder itself.

If we view proteins from the perspective of the folding energy landscape, the conformational dynamics vary from globular proteins with a well-defined global minimum to those that are present as highly dynamic ensembles of interconverting conformational states separated by low energy barriers, such as the structurally disordered proteins [70]. Structurally disordered proteins are prone to adopt different conformations (alter the conformational ensemble) in different environments and indeed structurally disordered regions show high conformational flexibility over different timescales and ranges of motion. Different conformational states are favored in interactions with different structural scaffolds and post-translational modifications are often involved in regulating conformational ensembles. As the structurally disordered proteins are characterized as conformational ensembles interconverting over a flattened energy landscape, mutations are likely to shift the conformational ensemble.

**Figure 2:**
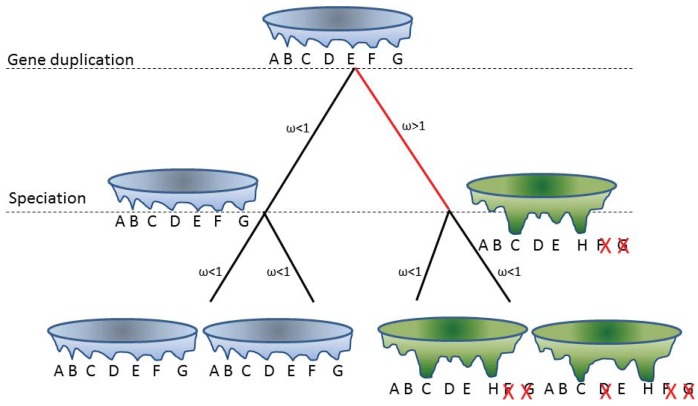
Evolution of an energy landscape and its conformational ensemble after gene duplication. At the root, the gene giving rise to the protein with the blue energy landscape resulting in conformations A to G is duplicated. At the next speciation event we can see that the two different gene copies have evolved along different trajectories. The blue copy at the speciation node has evolved under negative selection and resembles the ancient blue. The green copy at the speciation node has evolved under positive selection and of the original conformational ensemble, conformations F and G are no longer forming, but a new conformation, H, is forming. In addition, the equilibrium of the conformations is different in the blue *vs.* green energy landscapes. From the speciation node down to the extant sequences, blue is much conserved, while green although under negative selection, will lose conformation D, in one lineage. Analysis of the extant sequences would show that blue and green are structurally disordered homologs. However, although all these proteins are structurally disordered, the conformational ensembles differ between blue and green (while being the same within the blue copies, and very similar within the green copies.)

One of the mechanisms for generating novel or partitioned functions is through gene duplications/gene redundancy. It was recently shown that gene retention after gene duplication is higher for genes with many phosphorylation sites [71]. Structurally disordered proteins are enriched in phosphorylation sites and perhaps the thermodynamics of disorder in itself can provide an explanation. For globular structured proteins one main determinant for fixing a mutation is the effect of the mutation on the stability of the protein fold. Structurally disordered proteins are already less stable than the globular protein and exist as interconverting conformational ensembles. Therefore one might expect that these proteins will follow different rules. Here, a certain mutation may not abolish all conformations but simply a subset of the conformational ensemble. On shorter time scales, mutations that affect the equilibrium of the conformational ensemble can be regarded as influencing the function rather than the structure, while on longer time scales large changes in the conformational ensemble from a pair of gene duplicates may no longer overlap and can be regarded as changing the structure or fold. This would reflect a fold transition; a change from one fold or conformational ensemble into a distinctly different fold or conformational ensemble. Hence, structurally disordered proteins (proteins present as conformational ensembles) provide a mechanism for neostructuralization. An example of this concept is illustrated in Figure 2.

## 7. Designability of Structurally Disordered Proteins

Structurally disordered proteins are present as conformations of very low stability, distributed over a locally flat energy landscape. A mutation is likely to rearrange the conformational equilibrium and hence, mutations can be stabilizing, neutral, and destabilizing for different parts of the conformational ensemble at the same time. A mutation can alter the conformational ensemble, making a subset of conformations essentially unpopulated while functional conformations for which the mutation is stabilizing may gain population. This will result in a new energy landscape. If the new energy landscape is slightly less flat and has a few deeper wells, it could result in mutation driven conformational selection, which explains how structurally disordered proteins or regions can speed up the evolution of the protein structural landscape. Hence mutation driven conformation selection contributes to neostructuralization with different predominant conformations among homologs. Globular structured proteins that maintain their fold despite high sequence divergence have high designability (reviewed in [72]). Structurally disordered proteins evolve at elevated rates compared to many globular proteins [66–68], but does this mean that structural disorder has high designability with functional consequences or does it mean that most substitutions do not change the conformational ensemble significantly and are in fact functionally neutral? Can evolution of structurally disordered proteins provide a mechanism for neutral mutations to drive biological divergence [73]? Structurally disordered proteins are known to have a broad functional spectrum (reviewed in [74]), and this can lead to functional partitions after gene duplication. In a more subtle case, structurally disordered proteins can generate small changes in phenotype by a change in genotype that affects the conformational ensemble. If several conformational ensembles are altered in a small but cooperative manner, it could provide an underlying mechanism for structural divergence driving functional and phenotypic differences.

From an understanding of the evolutionary behavior of ordered and disordered proteins from a biophysical perspective comes the goal of modeling the evolution of proteins with more realistic models.

## 8. Modeling Evolution of Structurally Ordered Proteins

An overview of methods for modeling of the evolution of structurally ordered proteins has recently been described [24] and will only be summarized here. Two research trajectories have emerged that model the evolution of sequences in structurally ordered regions for evolutionary purposes. Retrospective analysis, particularly in the construction of phylogenetic trees [75–77] is one trajectory, where structural and biophysical considerations are viewed as an integral component of the evolution of proteins over long evolutionary distances and attempts have been made to replace purely statistical models that account for structure with the use of either a gamma distribution or a covarion process [78]. A second trajectory that has emerged is in the forward evolution of proteins, or sequence simulation constrained by a fold that does not vary [18,19].

For both of these trajectories, two classes of models are available, informational and physical models. In informational models, average interaction propensities extracted from PDB are summarized in matrices that reflect informational potentials [79,80]. These models can suffer from a lack of folding specificity [19,75–77]. An alternative is the use of models rooted in the physical principles of inter-atomic or inter-residue interaction. Because of the large number of calculations involved in both forward and retrospective evolutionary analysis, some degree of coarse-graining is necessary. The early physical coarse-grained models appear to be more specific than the informational potentials, but still have barriers to overcome, including a representation of side chains that leads to a properly packed hydrophobic core [19]. Research in these trajectories is ongoing.

## 9. Modeling Evolution of Structurally Disordered Proteins

One important trajectory will be to extend the models for structurally ordered regions to structurally disordered regions. Structurally disordered regions are functional in two key ways. Some structurally disordered regions become ordered upon binding and function as ordered regions [81]. In this case, the problem is simpler in that the proteins can be simulated as ordered while accounting in the model for the energy associated with the order to disorder transition. This will initially only approximate differences in the energy of this transition for different binding partners that is not reflected in differences in energy accounted for in the modeled ordered state. Nothing along these lines has yet been implemented.

A second class of disordered proteins are those that function as disordered regions [81]. To model such proteins, it will be important to uncover the sequence constraints on their disorder to be functional, as this will reflect a departure from neutrality in evolutionary rate. To the extent that this is a sequence rather than structural constraint, standard Markov Models will likely be appropriate [68]. One pitfall with Markov Models is that they generalize evolutionary properties that may be context dependent and better models are not conceivable without a better understanding of the evolutionary and biophysical properties of disordered regions.

In both cases, an important added constraint may be that the sequence in its unbound state is disordered rather than ordered. This constraint can be added to the model to select against mutations that would lead to a folded state. A random contacts model [82] could be implemented and a feature of this nature is implemented in IUPred, based upon an evaluation of the existence of favorable contacts for folding within the region [83].

## 10. Conclusions

As computational molecular biology and computational molecular evolution mature as fields, considerations of both the biophysical and the evolutionary attributes of proteins are increasingly being integrated. This coincides with an appreciation of the complexity of the biophysical chemistry of proteins in a cell, including the role of conformational ensembles, of post-translational modifications, of folding pathways, of protein kinetics, of protein complexes, and eventually of other cellular attributes, such as the role of chaperones. This is ultimately underpinned by an understanding of the energy landscape for a single sequence, and for homologous sequences linked through the mutational process. Simultaneously, protein structural and biophysical models will increasingly need to explicitly consider evolutionary processes as well in the field of structural bioinformatics. With these considerations, models will become more powerful (and slower) as the field moves forward.
